# Characteristics of Facial Asymmetry in Congenital Superior Oblique Palsy according to Trochlear Nerve Absence

**DOI:** 10.1155/2020/9476749

**Published:** 2020-12-17

**Authors:** Hee Kyung Yang, Sumin Jung, Taeg Keun WhangBo, Jeong-Min Hwang

**Affiliations:** ^1^Department of Ophthalmology, Seoul National University College of Medicine, Seoul National University Bundang Hospital, Seongnam, Republic of Korea; ^2^Computer Science, Gachon University, Seongnam, Republic of Korea; ^3^IT Department, Gachon University, Seongnam, Republic of Korea

## Abstract

**Methods:**

A total of 287 consecutive patients diagnosed with congenital SOP and 82 control subjects were included. Congenital SOP patients were grouped according to the presence (present group) or absence (absent group) of the trochlear nerve using thin-section high-resolution MRI of cranial nerves. We developed a computer-aided detection (CAD) system that could automatically analyze objective indices of facial asymmetry using frontal face photographs.

**Results:**

Of the 287 patients with congenital SOP, 60% of patients had ipsilateral trochlear nerve absence and superior oblique muscle (SO) hypoplasia (absent group), while the remaining 40% had a normal SO and trochlear nerve (present group). All but one objective indices related to facial asymmetry were significantly different between congenital SOP patients and controls (all *P* < 0.05). Among these features, the angle of nose deviation was significantly larger in the absent group compared to the present group (*P* < 0.001).

**Conclusion:**

Objective analysis of facial asymmetry using our novel CAD system was useful for identifying distinct features of congenital SOP. Deviation of the nose was more prominent in congenital SOP patients with trochlear nerve absence.

## 1. Introduction

Congenital superior oblique palsy (SOP) is one of the most common causes of ocular torticollis in children, of which the patient tilts his or her head to use both eyes together [1–3]. In children with torticollis, asymmetric development of the face is an irreversible but under-recognized complication of long-standing head tilt, especially if the head tilt is intermittent or mild [3–6]. Facial asymmetry is progressive if the head tilt persists in young children, and early strabismus surgery to correct the head tilt may help prevent facial asymmetry in congenital SOP [7]. Meanwhile, regarding the etiologic classification of congenital SOP, patients with an absent trochlear nerve may show more prominent head tilt and facial asymmetry compared to those with the presence of a trochlear nerve [8]. Therefore, successful treatment and prevention of facial asymmetry depend on an accurate diagnosis of the cause and careful monitoring of the degree of facial asymmetry, which may help determine the timing of intervention in patients with congenital SOP [9].

Facial asymmetry is usually assessed qualitatively by the subjective judgement of the observer. A few attempts have been made to quantify the degree of facial asymmetry objectively using several landmarks of anthropometric measurements on photographs, cephalometric assessment, and with the help of 3-dimensional analysis [2, 4, 9, 10]. However, there is a risk of radiation using cephalometric radiographs, and optical 3-dimensional surface analysis using a 3-camera fringe projection system is not easily accessible, time-consuming, and expensive [2, 4, 9, 10]. Besides, most of the studies are not validated in a large number of subjects and we do not know which asymmetry index is significantly related to perceived symmetry in various congenital and developmental situations related to facial growth. Therefore, a simple and reliable method to detect facial asymmetry using two-dimensional face photographs could be cost-effective and useful.

In this study, we developed an objective method to quantify the amount of facial asymmetry assisted by computer-aided automated feature extraction. Using this novel software, we aimed to determine if the asymmetry of facial characteristics differed among various etiologies of congenital SOP.

## 2. Materials and Methods

### 2.1. Subjects

A retrospective review of medical records was performed on 287 consecutive patients diagnosed with congenital SOP who underwent high-resolution thin-section magnetic resonance (MR) imaging at Seoul National University Bundang Hospital between November 2003 and October 2019. The subjects were divided into two groups according to MR image findings of the ipsilateral trochlear nerve; congenital SOP without a trochlear nerve (absent group) and congenital SOP with symmetric trochlear nerves on both sides (present group). Subjects with orthotropic or horizontal strabismus with no apparent head tilt, oblique muscle dysfunction nor any vertical strabismus were included as the control group.

Congenital SOP patients were included if they showed the typical signs of apparent underdepression and overelevation in adduction on the affected side, positive head-tilt test, large fusional amplitudes of vertical deviation, and/or a history or photographic evidence of long-standing strabismus or anomalous head posture dating back to infancy. Patients who had primary overaction of the inferior oblique muscle (IO) on the affected side, any evidence of acquired disease, a history of head or ocular trauma, or other potential causes such as plagiocephaly, skew deviation, or the ocular tilt reaction were excluded. All patients underwent a thin-section MRI at the brainstem level to clarify the presence of the trochlear nerve following the protocols introduced in our previous study [8, 11]. Approval to conduct this study was obtained from the Institutional Review Board of Seoul National University Bundang Hospital.

We noted patient characteristics, including gender, birth history, family history, initial signs/symptoms at presentation (chief complaint) such as head tilt, ocular deviation, or diplopia, age at onset, best-corrected visual acuity, cycloplegic refractive errors, and presence of amblyopia defined as a difference of two or more lines between monocular visual acuities and anisometropia >1.50 diopters. Extraocular movements and any incomitance were documented at the time of initial presentation and during follow-up examinations, including Bielschowsky's head-tilt test and ocular alignment with a prism cover test in six cardinal positions of gaze at distance in cooperative patients.

### 2.2. Computer-Aided Detection of Facial Asymmetry

We proposed a computer-aided detection (CAD) system that could automatically analyze objective indices of facial asymmetry. The proposed system consists of two steps: (i) image preprocessing with facial feature extraction and (ii) measurement of each facial asymmetry feature.

### 2.3. Extraction of Facial Indices

**Figure 1:**
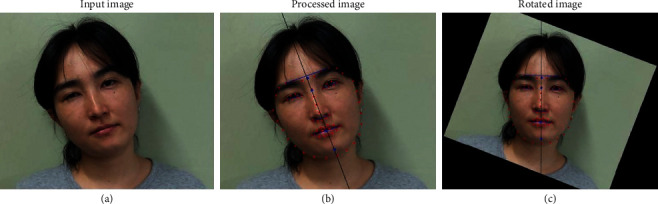
Automated analysis of facial asymmetry indices using computer-aided detection. (a) The original frontal face photograph is uploaded in the software. (b) The system automatically detects facial features of the eyes, nose, mouth, and facial outlines defined by 68 landmark points using the active appearance model (AAM). The degree of face tilt (°) is calculated by the angle of the line that passes between the landmark points of the facial midline (black line) in reference to the vertical line (angle of inclination 90°). (c) Rotation of the image is automatically processed according to the degree of face tilt.

First of all, the system automatically detects facial features such as the eyes, nose, mouth, and facial outlines from the frontal facial photograph of a patient and uses them as the basis for calculating facial asymmetry indices (Figure 1). Facial features of the eyes, nose, mouth, and facial outlines are defined by 68 landmark points using the active appearance model (AAM) [12]. The AAM is a vector-based algorithm that extracts facial features using a statistical model regarding the shape and texture information of an object based on principal component analysis [12]. It has been widely used in various applications such as face recognition, face modeling, and facial expression recognition [13].

### 2.4. Objective Measurement of Facial Asymmetry

**Figure 2:**
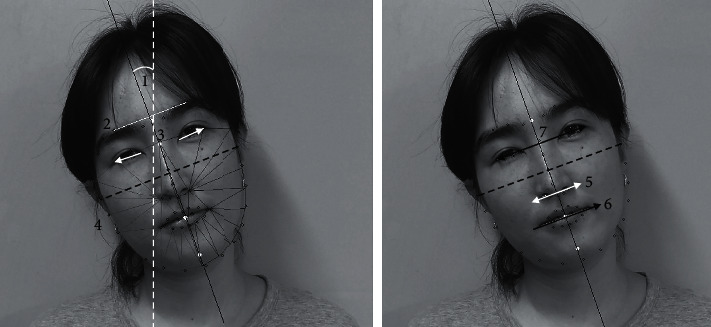
Seven indices of facial asymmetry automatically measured by the software. (1) Face tilt (°) is the angle between the vertical line (white dotted line) and the line that passes between the landmark points of the facial midline (black line). (2) Slope of eyebrows (°) is the angle between the line connecting both eyebrows (white line) and the perpendicular line to the facial midline (black dotted line). (3) Difference in slopes of eyes (°) is calculated by the difference in slopes of the lines passing through the eye corner points of each eye (white arrows). (4) Difference in hemifacial area (%) was calculated by the proportion of “absolute difference of left and right hemifacial area” to “total face area.” (5) Nose deviation (°) is the angle between the line connecting the corners of the nasal base (white double headed arrow) and the perpendicular line to the facial midline (black dotted line). (6) Slope of lips (°) is the angle between the line that passes through the corners of both lips (black arrow) and the perpendicular line to the facial midline (black dotted line). (7) Difference in slopes of eye and mouth (°) was calculated by the angle between the line connecting the center of each eye (black double-headed arrow) and the line that passes through the corners of both lips (black arrow). The center of each eye was calculated as the average coordinate of six landmark points on the upper and lower eyelids.

Seven indices of facial asymmetry were automatically extracted from frontal face photographs as follows: the degree of face tilt, slope of eyebrows, difference in slopes of eyes, difference in hemifacial area, nasal deviation, slope of lips, and difference in the slopes of eyes and mouth (Figure 2). Each feature is used as a variable for geometric calculation to estimate the degree of facial asymmetry.

The slope, width, and height of all facial asymmetry indices are calculated using two or more facial landmarks geometrically. To calculate the degree of face tilt, the system uses landmark points of four facial features, the eyebrows, eyes, mouth, and jaw, from previously extracted data. The facial midline is determined by these features using an algorithm of line fitting [14]. The angle between the facial midline and the vertical line is defined as the degree of face tilt.

Facial asymmetry indices are measured on the assumption that a patient's face is aligned without tilting. This is not true, particularly in patients with torticollis. Therefore, the system adjusts the value of all slopes based on the previously obtained degree of face tilt. Slope correction allows the system to calculate the value of each facial asymmetry feature, even when the patient's face is tilted in the image.

### 2.5. Statistical Analysis

Clinical characteristics and facial asymmetry indices were compared between patients with congenital superior oblique palsy with and without a trochlear nerve and controls using one-way ANOVA. All tests were performed using the SPSS version 25.0 software package (SPSS Inc., Chicago, IL, USA). A post hoc analysis was used to determine significant differences between two specific groups. Scheffe post hoc analysis was used when the groups met equal variance assumptions, while Dunnett T3 post hoc analysis was used when the equal variance was not assumed.

## 3. Results

### 3.1. Subjects' Characteristics

Finally, 287 patients with congenital SOP and 82 control subjects were included. Among the patients with congenital SOP, 173 (60%) showed ipsilateral absence of the trochlear nerve and SO hypoplasia (absent group), while the remainder (40%) showed symmetric trochlear nerves on both sides (present group). The clinical characteristics of congenital SOP patients and controls are summarized in Table 1. The mean age at examination, gender, cycloplegic refractive error, presence of anisometropia, and amblyopia were not significantly different between the three groups. In the control group, horizontal strabismus was found in 84% of subjects of which the majority was exotropia.

Early onset of head tilt before 1 year of age was significantly more frequent in the absent group compared to the present group in congenital SOP patients (*P*=0.005). The angle of hypertropia in primary gaze was larger in the absent group compared to the present group (*P* < 0.001).

### 3.2. Facial Asymmetry in Congenital Superior Oblique Palsy

Objective analysis of the seven indices of facial asymmetry was performed using frontal face photographs of congenital superior oblique palsy patients with and without a trochlear nerve and control subjects (Table 2).

All indices of facial asymmetry except the difference in the slopes of eyes were significantly different between congenital SOP patients and controls (all *P* < 0.05). Among the six indices that were significantly different between congenital SOP and controls, only the angle of nose deviation was significantly larger in the absent group compared to the present group (*P* < 0.001). No other feature, including the degree of face tilt, showed a significant difference between both groups of congenital SOP.

## 4. Discussion

In this study, we developed a novel automated CAD software to objectively assess the characteristics of facial asymmetry. Facial asymmetry features were significantly different in congenital SOP compared to controls. While most of the asymmetry indices were not significantly different according to the specific etiology of congenital SOP, the angle of nose deviation was larger in the absent group, which could be the most sensitive index of progressive facial asymmetry related to persistent head tilt.

Yi and Jang [15] found that facial asymmetry occurs more commonly with a deviated nose and suggested that a deviated nose might be a developmental defect caused by a discrepancy in the 2-side facial bone growth. In this study, nose deviation was the most obvious differentiating point between congenital SOP patients with versus without the trochlear nerve, which suggests a more prominent developmental defect of facial bone growth in congenital SOP patients without a trochlear nerve. This may probably be related to an earlier onset of head tilt, and early surgery could be helpful for congenital SOP patients with an absent trochlear nerve.

In spite of the rapid development in imaging technology, verification of the trochlear nerve is challenging because of its small diameter as well as its oblique course in the cisternal area [16]. A high-resolution and thin-section sequence using a 3 Tesla (*T*) MRI could classify the etiology of SOP according to the presence or absence of the trochlear nerve, and we have found some clinical differences between the two groups [8, 11, 17]. However, a 3T MRI may not always be available, and thin-section sequence imaging with 0.25 mm thickness requires a long scanning time. Therefore, any clues to predict the absence or presence of the trochlear nerve would be very useful, and the results of our study could add more to the existing clues [18, 19].

Head tilt in old photographs and facial asymmetry could be strong evidence of congenital SOP. However, there are some debate about the relationship between facial asymmetry and torticollis. Wilson and Hoxie [5] found contralesional hemifacial microsomia in most of the patients with congenital SOP. Paysee et al. [20] reported head tilt in 86% and facial asymmetry in 76% of patients with unilateral congenital SOP, while patients with acquired SOP showed head tilt in only 33% and none of them had facial asymmetry. In contrast, Velez et al. [21] evaluated three facial morphometric features in frontal photographs: the angle of inclination of each orbit, relative facial size, and facial angle. They concluded that facial asymmetry was not useful for distinguishing congenital SOP from acquired SOP or heterotopic rectus muscles.

Many algorithms have been proposed for automatic face recognition, most of which use Haar-like features that were introduced in the first real-time face detector, boosting the digital image features used in object recognition [22]. However, the detection capability of these algorithms is weakened when the image is rotated or the contrast has changed. In this study, we set facial landmark points that were initially detected in frontal face photographs using the AAM [12]. This method is based on shape and texture information of each landmark that is trained in advance with a large amount of face image data. A part having a texture most similar to each landmark can be searched to find the location of the landmark point more robustly than the existing algorithms [12]. In future work, higher performance can be expected by using machine learning for disease classification based on the results of asymmetric facial features.

There are some limitations in this study. First, we only obtained facial images of Asians. Obamiyi et al. [23] found radiographic differences such as the smallest mean cranial base and significantly larger *Y*-axis in the Chinese patients with temporomandibular joint disorders. Cheong and Lo [24] assumed that facial asymmetry might be more common in the normal Asian population than those in the Western countries. Further studies are necessary to reveal the differences in facial asymmetry according to different races. Second, all patients in this study had congenital SOP. Therefore, we could not be sure about the usefulness of this CAD software in other causes of facial asymmetry. Lastly, our software is based on 2-dimensional analysis. A 3-dimensional facial analysis may provide more precise information on developmental defects. Nevertheless, we could find some useful indices to differentiate congenital SOP patients with the absence of a trochlear nerve. In addition, our novel automated CAD software could avoid exposure to radiation hazard and save the time and cost for optical 3-dimensional surface analysis.

## 5. Conclusions

In conclusion, objective analysis of facial asymmetry using our novel CAD system was useful for identifying distinct features of congenital SOP. Deviation of the nose was more prominent in congenital SOP patients with the absence of a trochlear nerve.

## Figures and Tables

**Table 1:** Clinical characteristics of congenital superior oblique palsy patients with the absence of a trochlear nerve (absent group) and the presence of a trochlear nerve (present group) compared with controls.

	Absent group (*n* = 173)	Present group (*n* = 114)	Control (*n* = 82)	*P* value
Age at examination	21.7 ± 22.8	22.8 ± 20.1	16.9 ± 17.3	0.122^a^
Male gender	101 (58%)	68 (60%)	45 (55%)	0.792^c^
Cycloplegic refractive errors (*D*)	−0.23 ± 2.03	−0.57 ± 2.52	−0.37 ± 2.67	0.488^a^
Anisometropia >1.50 (*D*)	9 (5%)	10 (9%)	11(13%)	0.080^c^
Amblyopia	15 (9%)	2 (2%)	6 (7%)	0.054^c^
Horizontal strabismus	**86 (50%)**	**67 (59%)**	**69 (84%)**	<0.001^a^
Exotropia	80/86 (93%)	57/67 (85%)	52/69 (75%)	—
Esotropia	6/86 (7%)	10/67 (15%)	17/69 (25%)	—
Unilateral SOP	171 (99%)	114 (100%)	—	0.255^c^
Right	91 (53%)	52 (46%)	—	0.235^c^
Early onset of head tilt^d^	**70 (41%)**	**28 (25%)**	—	0.005^b^
Hypertropia (PD)	**14.2** **±** **8.6**	**10.3** **±** **6.8**	—	<0.001^c^

y = years; *D* = diopters; SOP = superior oblique palsy; PD = prism diopters; significant factors are expressed in bold characters; ^a^*P* value by one-way ANOVA; ^b^*P* value by the independent *t*-test; ^c^*P* value by the Pearson chi-square test; ^d^patients who had reliable data at the time of onset of definite head tilt before 1 year of age.

**Table 2:** Objective measurement of facial asymmetry features in congenital superior oblique palsy patients with the absence of a trochlear nerve (absent group) or presence of a trochlear nerve (present group) compared with controls.

	Absent group (1) (*n* = 173)	Present group (2) (*n* = 114)	Control (3) (*n* = 82)	*P* value^a^	Post hoc
Face tilt (°)	5.36 ± 5.00 (0–26.30)	4.23 ± 3.69 (0–20.50)	2.17 ± 1.99 (0–12.65)	<0.001	1, 2 > 3
Slope of eyebrows (°)	3.92 ± 2.68 (0–11.81)	3.34 ± 2.50 (0–10.59)	1.87 ± 1.50 (0–8.08)	<0.001	1, 2 > 3
Difference in slopes of eyes (°)	9.51 ± 5.51 (0–22.62)	7.64 ± 5.58 (0–24.45)	11.07 ± 5.05 (0–26.73)	<0.001	1, 3 > 2
Difference in hemifacial area^b^ (%)	3.73 ± 3.58 (0.03–21.52)	3.28 ± 3.49 (0–17.84)	2.58 ± 2.09 (0.05–10.57)	0.034	1, 2 > 3
Nose deviation (°)	3.91 ± 2.91 (0–12.34)	2.87 ± 2.39 (0–11.28)	2.02 ± 1.53 (0–7.80)	<0.001	1 > 2 > 3
Slope of lips (°)	4.04 ± 3.61 (0–17.99)	3.63 ± 3.01 (0–14.15)	2.10 ± 1.99 (0–12.22)	<0.001	1, 2 > 3
Difference in slopes of eye and mouth (°)	0.04 ± 0.03 (0–0.22)	0.03 ± 0.02 (0–0.13)	0.02 ± 0.02 (0–0.08)	<0.001	1, 2 > 3

Values are presented as mean ± standard deviation. ^a^*P* value by one-way ANOVA. Post hoc test was performed by Dunnett T3. ^b^Proportion of “absolute difference of left and right hemifacial area” to “total face area.”

## Data Availability

Data supporting the findings of the current study are available from the corresponding author upon reasonable request.
